# 10*H*-Phenothia­zine 5-oxide

**DOI:** 10.1107/S1600536810047914

**Published:** 2010-11-24

**Authors:** Rui-Fang Jin, Kai Yu, Shi-Yao Yang, Rong-Bin Huang

**Affiliations:** a Department of Chemistry, College of Chemistry and Chemical Engineering, Xiamen University, Xiamen 361005, People’s Republic of China

## Abstract

In the title compound, C_12_H_9_NOS, the sulfoxide O atom is disordered over two sites with occupancies of 0.907 (4) and 0.093 (4). The dihedral angle betweeen the two aromatic rings is 18.40 (14)°. Different types of supramolecular interactions including inter­molecular N—H⋯O hydrogen bonds and π–π contacts [centroid–centroid distances = 3.9096 (16) and 4.1423 (16) Å] between the aromatic rings of symmetry-related mol­ecules are observed in the crystal structure.

## Related literature

For *N*-aryl­phenothia­zine structures, see: Chu & Van der Helm (1974 ▶, 1975 ▶, 1976 ▶) and for *N*-aryl­phenothia­zine oxide structures, see: Chu *et al.* (1985 ▶), Wang *et al.* (2009 ▶). For a dioxophenothia­zinium cation co-crystallized with terephthalate trihydrate, see: Zhu *et al.* (2007 ▶).
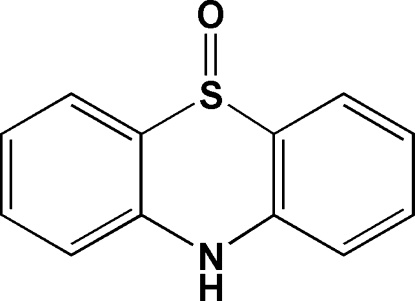


## Experimental

### 

#### Crystal data

C_12_H_9_NOS
*M* *_r_* = 215.26
Monoclinic, 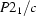

*a* = 6.4482 (4) Å
*b* = 7.6610 (5) Å
*c* = 22.0956 (14) Å
β = 110.466 (2)°
*V* = 1022.62 (11) Å^3^
*Z* = 4
Mo *K*α radiation
μ = 0.29 mm^−1^
*T* = 297 K
0.50 × 0.50 × 0.40 mm

#### Data collection

Bruker APEX area-detector diffractometer
Absorption correction: multi-scan (*SADABS*; Bruker, 2002 ▶) *T*_min_ = 0.871, *T*_max_ = 0.895
7632 measured reflections
2361 independent reflections
1962 reflections with *I* > 2σ(*I*)
*R*_int_ = 0.029

#### Refinement

*R*[*F* ^2^ > 2σ(*F* ^2^)] = 0.063
*wR*(*F* ^2^) = 0.175
*S* = 1.04
2361 reflections
146 parameters
6 restraints
H-atom parameters constrained
Δρ_max_ = 0.44 e Å^−3^
Δρ_min_ = −0.19 e Å^−3^

### 

Data collection: *SMART* (Bruker, 2002 ▶); cell refinement: *SAINT* (Bruker, 2002 ▶); data reduction: *SAINT*; program(s) used to solve structure: *SHELXS97* (Sheldrick, 2008 ▶); program(s) used to refine structure: *SHELXL97* (Sheldrick, 2008 ▶); molecular graphics: *DIAMOND* (Brandenburg, 2010) ▶; software used to prepare material for publication: *publCIF* (Westrip, 2010 ▶).

## Supplementary Material

Crystal structure: contains datablocks I, global. DOI: 10.1107/S1600536810047914/si2310sup1.cif

Structure factors: contains datablocks I. DOI: 10.1107/S1600536810047914/si2310Isup2.hkl

Additional supplementary materials:  crystallographic information; 3D view; checkCIF report

## Figures and Tables

**Table 1:** Hydrogen-bond geometry (Å, °)

*D*—H⋯*A*	*D*—H	H⋯*A*	*D*⋯*A*	*D*—H⋯*A*
N10—H10*A*⋯O5^i^	0.86	2.10	2.856 (3)	146

Symmetry code: (i) 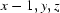.

**Fig. 1.**
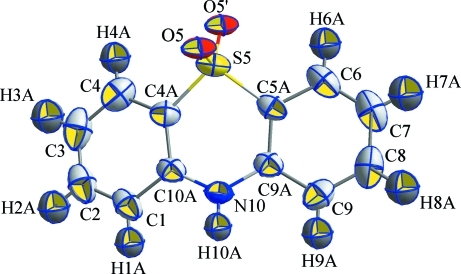
Thermal ellipsoid plot of I. Displacement ellipsoids are drawn at the 50% probabability level.

**Fig. 2.**
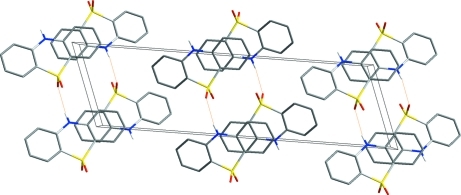
A perspective view of the crystal structure of I. Hydrogen atoms have been omitted for clarity.

## supplementary crystallographic information

### Comment

The crystal structures of *N*-arylphenothiazine (Chu & Van der Helm, 1974, 1975, 1976), *N*-arylphenothiazine oxides (Chu *et al.*, 1985; Wang *et al.*, 2009) and dioxide (Zhu *et al.*, 2007) have been reported, yet that of phenothiazine or its oxide has not been reported. The title compound (I) was obtained by the oxidation of phenothiazine in THF solution in air.

In the structure of I (Fig. 1), the sulfoxide O atom is disordered over two sites and the occupancy factors are 0.907 (4) (boat-axial S—O) and 0.093 (4) (boat-equatorial S—O). The same disorder in 10-acetyl-10*H*-phenothiazine 5-oxide was reported recently (Wang *et al.*, 2009). The weighted average S—O distance of 1.471 Å in I is comparable to 1.466 Å in 10-acetyl-10*H*-phenothiazine 5-oxide, 1.498 (2) Å in 10-methylphenothiazine 5-oxide, and longer than 1.446 Å for dioxophenothiazinium cation (Zhu *et al.* 2007). The significantly shorter N—C distances in I than those in other *N*-arylphenothiazines or oxides are due to N—H instead of *N*-aryl groups (see the following table). For the same reason the dihedral angle betweeen the two benzene rings 18.40 (14) ° in I is smaller than those in the other compounds.

N—C (Å) substituent (reference)

1.365 (3), 1.368 (3) H (this work)

1.402 (2), 1.455 (5) methyl (Chu & Van der Helm, 1974)

1.406 (4), 1.427 (4) ethyl (Chu & Van der Helm, 1975)

1.410 (2), 1.414 (2) isopropyl (Chu & Van der Helm, 1976)

1.428 (2), 1.436 (2) acetyl (Wang *et al.*, 2009)

1.409 (3), 1.409 (3) 2-dimethylammonium-propyl (Zhu *et al.* 2007)

In the crystal structure (Fig. 2), intermolecular interactions N—H···O hydrogen bond and π–π contacts between the aromatic rings [centroid to centroid distances = 3.9096 (16) and 4.1423 (16) Å] of symmetry-related molecules are observed.

### Experimental

A mixture of 1,3,5-benzenetricarboxylic acid (0.5 mmol) and phenothiazine (0.5 mmol) was dissolved in 10 ml THF. The solution changed from colorless to red in air in several hours. Brown crystals were obtained by slow evaporation for about 4 days at room temperature.

### Refinement

The aromatic H atoms were generated geometrically (C—H 0.93, N—H 0.86 Å) and were allowed to ride on their parent atoms in the riding model approximations, with their temperature factors set to 1.2 times those of the parent atoms. The position of the oxygen atom is refined at two sites, with occupancy factors of 0.907 (4) and 0.093 (4).

### Crystal data

**Table:**

C_12_H_9_NOS	*F*(000) = 448
*M**_r_* = 215.26	*D*_x_ = 1.398 Mg m^−^^3^
Monoclinic, *P*2_1_/*c*	Mo *K*α radiation, λ = 0.71073 Å
Hall symbol: -P 2ybc	Cell parameters from 3079 reflections
*a* = 6.4482 (4) Å	θ = 2.7–27.3°
*b* = 7.6610 (5) Å	µ = 0.28 mm^−^^1^
*c* = 22.0956 (14) Å	*T* = 297 K
β = 110.466 (2)°	Block, brown
*V* = 1022.62 (11) Å^3^	0.50 × 0.50 × 0.40 mm
*Z* = 4	

### Data collection

**Table:**

Bruker APEX area-detector diffractometer	2361 independent reflections
Radiation source: fine-focus sealed tube	1962 reflections with *I* > 2σ(*I*)
graphite	*R*_int_ = 0.029
φ and ω scan	θ_max_ = 28.6°, θ_min_ = 2.0°
Absorption correction: multi-scan (*SADABS*; Bruker, 2002)	*h* = −8→8
*T*_min_ = 0.871, *T*_max_ = 0.895	*k* = −9→9
7632 measured reflections	*l* = −28→29

### Refinement

**Table:**

Refinement on *F*^2^	Primary atom site location: structure-invariant direct methods
Least-squares matrix: full	Secondary atom site location: difference Fourier map
*R*[*F*^2^ > 2σ(*F*^2^)] = 0.063	Hydrogen site location: inferred from neighbouring sites
*wR*(*F*^2^) = 0.175	H-atom parameters constrained
*S* = 1.04	*w* = 1/[σ^2^(*F*_o_^2^) + (0.098*P*)^2^ + 0.4384*P*] where *P* = (*F*_o_^2^ + 2*F*_c_^2^)/3
2361 reflections	(Δ/σ)_max_ < 0.001
146 parameters	Δρ_max_ = 0.44 e Å^−^^3^
6 restraints	Δρ_min_ = −0.19 e Å^−^^3^

### Special details

**Table:**

Geometry. All e.s.d.'s (except the e.s.d. in the dihedral angle between two l.s. planes) are estimated using the full covariance matrix. The cell e.s.d.'s are taken into account individually in the estimation of e.s.d.'s in distances, angles and torsion angles; correlations between e.s.d.'s in cell parameters are only used when they are defined by crystal symmetry. An approximate (isotropic) treatment of cell e.s.d.'s is used for estimating e.s.d.'s involving l.s. planes.
Refinement. Refinement of *F*^2^ against ALL reflections. The weighted *R*-factor *wR* and goodness of fit *S* are based on *F*^2^, conventional *R*-factors *R* are based on *F*, with *F* set to zero for negative *F*^2^. The threshold expression of *F*^2^ > σ(*F*^2^) is used only for calculating *R*-factors(gt) *etc*. and is not relevant to the choice of reflections for refinement. *R*-factors based on *F*^2^ are statistically about twice as large as those based on *F*, and *R*- factors based on ALL data will be even larger.

### Fractional atomic coordinates and isotropic or equivalent isotropic displacement parameters (Å^2^)

**Table:**

	*x*	*y*	*z*	*U*_iso_*/*U*_eq_	Occ. (<1)
S5	0.42382 (10)	0.16598 (9)	0.58407 (3)	0.0511 (3)	
O5	0.5476 (3)	0.3348 (3)	0.60011 (10)	0.0515 (6)	0.907 (4)
O5'	0.537 (2)	0.0431 (17)	0.5773 (6)	0.024 (4)	0.093 (4)
N10	−0.0212 (3)	0.2763 (3)	0.59516 (10)	0.0465 (5)	
H10A	−0.1256	0.3363	0.6009	0.056*	
C1	−0.1959 (5)	0.3230 (3)	0.48155 (14)	0.0563 (7)	
H1A	−0.3197	0.3677	0.4885	0.068*	
C2	−0.1951 (6)	0.3105 (4)	0.42027 (16)	0.0695 (9)	
H2A	−0.3182	0.3472	0.3859	0.083*	
C3	−0.0141 (7)	0.2438 (4)	0.40843 (15)	0.0746 (10)	
H3A	−0.0169	0.2332	0.3662	0.090*	
C4A	0.1721 (4)	0.2076 (3)	0.52193 (12)	0.0459 (6)	
C4	0.1689 (6)	0.1936 (4)	0.45877 (15)	0.0624 (8)	
H4A	0.2917	0.1500	0.4509	0.075*	
C5A	0.3231 (4)	0.1236 (3)	0.64649 (12)	0.0458 (6)	
C6	0.4605 (5)	0.0291 (4)	0.69942 (15)	0.0621 (8)	
H6A	0.5904	−0.0202	0.6979	0.075*	
C7	0.4058 (6)	0.0086 (4)	0.75312 (16)	0.0750 (9)	
H7A	0.4974	−0.0554	0.7880	0.090*	
C8	0.2155 (7)	0.0822 (4)	0.75575 (15)	0.0725 (9)	
H8A	0.1807	0.0699	0.7930	0.087*	
C9A	0.1255 (4)	0.1936 (3)	0.64756 (12)	0.0433 (5)	
C9	0.0752 (5)	0.1739 (4)	0.70411 (15)	0.0592 (7)	
H9A	−0.0533	0.2232	0.7067	0.071*	
C10A	−0.0122 (4)	0.2694 (3)	0.53439 (12)	0.0429 (5)	

### Atomic displacement parameters (Å^2^)

**Table:**

	*U*^11^	*U*^22^	*U*^33^	*U*^12^	*U*^13^	*U*^23^
S5	0.0328 (4)	0.0552 (4)	0.0649 (5)	0.0046 (2)	0.0165 (3)	−0.0057 (3)
O5	0.0265 (9)	0.0631 (13)	0.0647 (13)	−0.0072 (8)	0.0156 (9)	−0.0056 (9)
O5'	0.024 (4)	0.025 (4)	0.025 (4)	0.0011 (10)	0.0089 (16)	−0.0008 (10)
N10	0.0303 (9)	0.0531 (12)	0.0573 (13)	0.0056 (9)	0.0166 (9)	0.0031 (10)
C1	0.0421 (14)	0.0503 (14)	0.0633 (17)	−0.0057 (11)	0.0018 (12)	0.0081 (12)
C2	0.067 (2)	0.0634 (18)	0.0589 (18)	−0.0144 (15)	−0.0016 (15)	0.0080 (14)
C3	0.096 (3)	0.073 (2)	0.0481 (17)	−0.026 (2)	0.0172 (17)	−0.0066 (15)
C4A	0.0401 (13)	0.0447 (12)	0.0511 (14)	−0.0055 (10)	0.0139 (11)	−0.0057 (10)
C4	0.0675 (19)	0.0626 (17)	0.0614 (17)	−0.0154 (14)	0.0279 (15)	−0.0152 (14)
C5A	0.0364 (12)	0.0416 (12)	0.0532 (14)	−0.0010 (10)	0.0077 (10)	−0.0025 (10)
C6	0.0524 (16)	0.0517 (15)	0.0674 (18)	0.0074 (12)	0.0024 (13)	0.0060 (13)
C7	0.080 (2)	0.0612 (19)	0.063 (2)	−0.0008 (17)	−0.0001 (17)	0.0118 (15)
C8	0.095 (3)	0.0701 (19)	0.0504 (17)	−0.0125 (18)	0.0227 (17)	0.0062 (14)
C9A	0.0352 (12)	0.0415 (12)	0.0502 (14)	−0.0050 (9)	0.0113 (10)	−0.0005 (10)
C9	0.0559 (17)	0.0644 (17)	0.0630 (17)	−0.0101 (13)	0.0278 (14)	−0.0038 (13)
C10A	0.0333 (11)	0.0391 (11)	0.0526 (14)	−0.0056 (9)	0.0101 (10)	0.0009 (10)

### Geometric parameters (Å, °)

**Table:**

S5—O5'	1.233 (13)	C4A—C10A	1.393 (3)
S5—O5	1.496 (2)	C4A—C4	1.393 (4)
S5—C5A	1.748 (3)	C4—H4A	0.9300
S5—C4A	1.750 (3)	C5A—C9A	1.390 (3)
N10—C10A	1.365 (3)	C5A—C6	1.397 (4)
N10—C9A	1.368 (3)	C6—C7	1.360 (5)
N10—H10A	0.8600	C6—H6A	0.9300
C1—C2	1.359 (5)	C7—C8	1.370 (5)
C1—C10A	1.403 (3)	C7—H7A	0.9300
C1—H1A	0.9300	C8—C9	1.376 (5)
C2—C3	1.380 (5)	C8—H8A	0.9300
C2—H2A	0.9300	C9A—C9	1.404 (4)
C3—C4	1.364 (5)	C9—H9A	0.9300
C3—H3A	0.9300		
			
O5'—S5—O5	113.5 (6)	C4A—C4—H4A	120.0
O5'—S5—C5A	110.7 (6)	C9A—C5A—C6	120.1 (3)
O5—S5—C5A	106.75 (12)	C9A—C5A—S5	122.5 (2)
O5'—S5—C4A	118.1 (6)	C6—C5A—S5	117.0 (2)
O5—S5—C4A	107.46 (12)	C7—C6—C5A	120.5 (3)
C5A—S5—C4A	98.86 (12)	C7—C6—H6A	119.7
C10A—N10—C9A	124.1 (2)	C5A—C6—H6A	119.7
C10A—N10—H10A	118.0	C6—C7—C8	119.9 (3)
C9A—N10—H10A	118.0	C6—C7—H7A	120.0
C2—C1—C10A	120.8 (3)	C8—C7—H7A	120.0
C2—C1—H1A	119.6	C7—C8—C9	120.9 (3)
C10A—C1—H1A	119.6	C7—C8—H8A	119.5
C1—C2—C3	120.8 (3)	C9—C8—H8A	119.5
C1—C2—H2A	119.6	N10—C9A—C5A	122.1 (2)
C3—C2—H2A	119.6	N10—C9A—C9	119.8 (2)
C4—C3—C2	119.9 (3)	C5A—C9A—C9	118.2 (2)
C4—C3—H3A	120.1	C8—C9—C9A	120.2 (3)
C2—C3—H3A	120.1	C8—C9—H9A	119.9
C10A—C4A—C4	120.6 (3)	C9A—C9—H9A	119.9
C10A—C4A—S5	121.9 (2)	N10—C10A—C4A	122.7 (2)
C4—C4A—S5	117.2 (2)	N10—C10A—C1	119.6 (2)
C3—C4—C4A	120.1 (3)	C4A—C10A—C1	117.7 (3)
C3—C4—H4A	120.0		
			
C10A—C1—C2—C3	0.3 (4)	C5A—C6—C7—C8	−0.6 (5)
C1—C2—C3—C4	−1.6 (5)	C6—C7—C8—C9	1.5 (5)
O5'—S5—C4A—C10A	145.5 (7)	C10A—N10—C9A—C5A	13.3 (4)
O5—S5—C4A—C10A	−84.5 (2)	C10A—N10—C9A—C9	−165.2 (2)
C5A—S5—C4A—C10A	26.3 (2)	C6—C5A—C9A—N10	−175.3 (2)
O5'—S5—C4A—C4	−40.9 (7)	S5—C5A—C9A—N10	11.0 (3)
O5—S5—C4A—C4	89.1 (2)	C6—C5A—C9A—C9	3.2 (4)
C5A—S5—C4A—C4	−160.1 (2)	S5—C5A—C9A—C9	−170.48 (19)
C2—C3—C4—C4A	0.8 (5)	C7—C8—C9—C9A	0.0 (5)
C10A—C4A—C4—C3	1.3 (4)	N10—C9A—C9—C8	176.2 (3)
S5—C4A—C4—C3	−172.4 (2)	C5A—C9A—C9—C8	−2.3 (4)
O5'—S5—C5A—C9A	−151.4 (7)	C9A—N10—C10A—C4A	−13.6 (4)
O5—S5—C5A—C9A	84.6 (2)	C9A—N10—C10A—C1	165.2 (2)
C4A—S5—C5A—C9A	−26.7 (2)	C4—C4A—C10A—N10	176.3 (2)
O5'—S5—C5A—C6	34.7 (7)	S5—C4A—C10A—N10	−10.4 (3)
O5—S5—C5A—C6	−89.3 (2)	C4—C4A—C10A—C1	−2.5 (4)
C4A—S5—C5A—C6	159.4 (2)	S5—C4A—C10A—C1	170.87 (18)
C9A—C5A—C6—C7	−1.8 (4)	C2—C1—C10A—N10	−177.1 (2)
S5—C5A—C6—C7	172.2 (2)	C2—C1—C10A—C4A	1.7 (4)

### Hydrogen-bond geometry (Å, °)

**Table:**

*D*—H···*A*	*D*—H	H···*A*	*D*···*A*	*D*—H···*A*
N10—H10A···O5^i^	0.86	2.10	2.856 (3)	146

Symmetry codes: (i) *x*−1, *y*, *z*.
